# The different expression of caspase-1 in HBV-related liver disease and acts as a biomarker for acute-on-chronic liver failure

**DOI:** 10.1186/s12876-019-1064-3

**Published:** 2019-08-20

**Authors:** Xiangying Zhang, Peiling Dong, Lin Xu, Yuan Tian, Huayin Sun, Hongbo Shi, Zhongping Duan, Liyan Chen, Feng Ren

**Affiliations:** 1 Beijing YouAn Hospital, Capital Medical University, Beijing, 100069 China; 2 The 2nd Affiliated Hospital of Harbin Medical University, Harbin, 150001 China

**Keywords:** Chronic hepatitis B, Acute-on-chronic liver failure, Inflammation, Biomarkers

## Abstract

**Background:**

Caspase-1 is an evolutionarily conserved enzyme that proteolytically cleaves the precursors of the inflammatory cytokines interleukin 1β and interleukin 18. However, the role of caspase-1 in determining the severity of acute-on-chronic liver failure (ACLF) has yet to be elucidated. We evaluated the expression levels of caspase-1 in HBV-related liver disease and assessed its utility as a biomarker predicting the severity of ACLF.

**Methods:**

The gene, protein and activity levels of caspase-1 were measured in the liver and/or serum of subjects with HBV-related disease. We also analysed the correlation between the expression levels of caspase-1 and liver injury of ACLF.

**Results:**

Compared with the values observed in normal subjects, the relative caspase-1 mRNA and protein levels in livers were decreased in patients with CHB, LC, and HCC but increased in those with ACLF; moreover, ACLF patients had the lowest serum level and hepatic activity of caspase-1 among the five groups. The serum caspase-1 levels in ACLF patients showed a negative correlation with total serum bilirubin and a positive correlation with serum total protein and albumin. Importantly, the serum caspase-1 levels in the surviving group with ACLF were higher than those in the non-surviving group and showed different dynamic trends. Analyses of the area under the receiver operating characteristic curve indicated that caspase-1 (AUC = 0.84, AUC of MELD score = 0.72) may be a useful marker for independently predicting ACLF.

**Conclusion:**

Caspase-1 is a potential non-invasive biomarker of disease progression and prognosis in ACLF.

## Background

Worldwide, 257 million persons, or 3.5% of the population, were living with chronic HBV infection in 2015. The African and Western Pacific regions accounted for 68% of those infected [1]. The overall infection rate of the Chinese population is 6.1%, accounting for approximately 1/3 of HBV cases worldwide [2]. Decompensated liver cirrhosis (LC), liver failure and hepatocellular carcinoma (HCC) caused by HBV infection cause approximately 900,000 deaths per year [3]. Acute exacerbation of CHB infection is not uncommon, with a cumulative incidence of 10–30% every year [4]. Acute-on-chronic liver failure (ACLF) is a common pattern of end-stage liver disease that occurs frequently in patients with chronic hepatitis B infection, causing a large burden of disease as it progresses rapidly [5]. Since the treatment options are limited, the outcome is generally poor. Liver transplantation is sometimes used in ACLF patients, but this procedure is costly and has variable success rates. Early intervention can improve survival, and the recognition of this effect has creased new demand for early diagnostic and prognostic markers.

The inflammasomes, which are multiprotein complexes involved in the assembly of pattern recognition receptors (PRRs) in the cytoplasm, are an important part of the natural immune system. The inflammasomes can recognize pathogen-associated molecular patterns (PAMPs) and host-derived risk signalling molecules (DAMPs), recruiting and activating the pro-inflammatory protein cysteinyl aspartate specific protease (caspase)-1. Activated caspase-1 cleaves precursors of IL-1β and IL-18, producing corresponding mature cytokines and pyroptosis; pyroptosis, in turn, leads to cell lysis and the release of cytoplasmic contents to the outside of the cell, causing inflammation [6]. Therefore, caspase-1 is a key inflammatory mediator driving the host response to infection, injury and disease.

Caspase-1 plays an important role in the pathogenesis of various liver diseases in vivo or in vitro, not only as an inflammatory mediator but also as a hepatocyte apoptosis mediator. The activation of caspase-1 is strongly involved in Fas-mediated apoptosis and TNF-α-induced hepatocyte injury [7, 8], and inhibition of caspase-1 activity substantially suppresses Fas-induced death [9]. Necrotic liver cells cause eosinophil pyroptosis (a programmed inflammatory form of cell death that has been reported to occur in macrophages and DCs and CD4+ cells. IL-1β and IL-18 secretion, degranulation, and cell death can be blocked by caspase-1 inhibitors, showing that all of these processes are indeed caspase-1 dependent [10]. There are also studies demonstrating that IL-18 can also be secreted from FasL-stimulated macrophages via a caspase-1-independent pathway and contribute to acute liver injury in mice [11]. Our studies showed that caspase-1 activation favoured the development of a pro-inflammatory response through a glycogen synthase kinase 3β-dependent mechanism and led to liver tissue damage in mouse ALF induced by D-galactosamine (D-GalN)/ lipopolysaccharide (LPS) [12]. Testing the role of IL-1β formation through caspase-1 in acetaminophen-induced hepatic inflammation and liver injury, C57BL/6 mice were treated with the pan-caspase inhibitor Z-VD-fmk to block the inflammasome-mediated maturation of IL-1β during APAP overdose. The observed effect was activation of caspase-1, and the inflammasome contributed to the formation of pro-inflammatory mediators after APAP [13]. However, Qian studied the role of caspase-1 inactivation in promoting adaptive responses to oxidative stress and, more specifically, in limiting reactive oxygen species production and damage in cells and tissues where IL-1β and IL-18 are not highly expressed [14].

However, the role of inflammasomes in HBV-related liver disease remains largely obscure, and studies of caspase-1 in ACLF caused by acute exacerbation of CHB are scarce. This study aimed to demonstrate that caspase-1 is elaborately regulated in HBV-related ACLF, LC and HCC patients, which may be a novel biomarker to forecast ACLF occurrence and prognosis due to acute exacerbation of CHB infection.

## Methods

### Study subjects and human sample collection

Serum samples were collected from 80 patients with HBV-related hepatocellular cancer (HCC), 62 patients with HBV-related liver cirrhosis (LC), 126 patients with HBV-related ACLF (including 30 survivors and 41 non-survivors), 101 patients with chronic hepatitis B (CHB) and 69 healthy subjects (the clinical characteristics and details of all subjects are described in Tables 1 and 2). Liver samples were collected from 20 patients with HBV-related HCC, 21 patients with HBV-related LC, 19 patients with HBV-related ACLF, 12 patients with chronic hepatitis B (CHB) and 10 normal liver samples. Normal liver specimens of 8 patients with hepatic resection for liver transplantation were collected. CHB samples were obtained from the livers of 12 patients undergoing liver puncture biopsy. LC, HCC and ACLF liver samples were obtained from the livers of patients with HBV infection undergoing liver transplantation. The diagnostic standard of HBV-related ACLF, HBV-related LC, HBV-related HCC and CHB fulfilled the definition set by the Asian Pacific Association for the Study of the Liver Working Party [5, 15].

**Table 1:** Demographic and clinical characteristics of the different serum study groups

Parameters	Control (*n* = 69)	CHB (*n* = 101)	LC (*n* = 62)	HCC (*n* = 80)	ACLF (*n* = 126)	*P* value
Age (years)	39.3 ± 6.3	40.3 ± 7.8	47.4 ± 9.2	51.1± 10.5	45.9 ± 9.8	0.066
Gender (male/female)	37/32	58/43	34/28	51/29	82/44	0.342
Alanine aminotransferase (U/L)	24.6 ± 17.2	98.8 ± 67.7	70.8 ± 26.1	63.1 ± 62.3	81.4 ± 59.9	0.008
Aspartate aminotransferase (U/L)	35.8 ± 6.4	101.1 ± 30.2	88.7 ± 19.5	77.6 ± 23.9	111.4 ± 30.1	< 0.001
Total bilirubin (μmol/l)	14.2 ± 3.9	59.7 ± 27.4	16.5 ± 8.8	21.9 ± 10.1	336.2 ± 87.3	< 0.001
Albumin (g/L)	45.6 ± 5.3	40.7 ± 5.7	38.5 ± 7.6	39.0 ± 8.3	32.4 ± 9.9	< 0.001
HBsAg positive (n)	0	101	62	80	126	–
HBV DNA (log_10_ copies/mL)	0	7.11 ± 3.9	6.37 ± 4.5	6.01 ± 2.9	5.87 ± 2.3	–
Creatinine (μmol/L)	74 ± 44.7	68 ± 30.6	65 ± 19.1	67 ± 52.4	63 ± 40.8	0.007
Prothrombin time (s)	10.8 ± 1.6	12.8 ± 4.0	17.9 ± 8.3	15.4 ± 7.1	25.2 ± 10.5	< 0.001
International normalized ratio	0.98 ± 0.12	1.17 ± 0.2	1.31 ± 0.22	1.25 ± 0.31	2.17 ± 0.47	< 0.001
IL-1β(pg/ml)	5.14 ± 2.64	6.81 ± 4.06	4.37 ± 2.99	5.55 ± 5.39	1.39 ± 0.82	< 0.001
IL-18(pg/m)	9.11 ± 2.66	17.61 ± 19.04	34.21 ± 33.78	50.14 ± 41.02	303.09 ± 274.54	< 0.001

*Abbreviations*: *CHB* chronic hepatitis B, *LC* liver cirrhosis, *HCC* hepatocellular carcinoma, *ACLF* acute-on-chronic liver failure, *IL-1β* interleukin-1β, *IL-18* interleukin-8

**Table 2:** Clinical features of ACLF patients based on different outcomes

Parameters	Survivors (*n* = 30)	Non-survivors (*n* = 41)	*P* value
Age (years)	43.08 ± 9.7	46.10 ± 11.3	0.131
Gender (male/female)	18/12	26/15	0.293
Alanine aminotransferase (U/L)	444.9 ± 58.3	490.3 ± 66.2	0.614
Aspartate aminotransferase (U/L)	501.3 ± 87.6	536.1 ± 74.6	0.592
Total bilirubin (μmol/l)	332.8 ± 100.3	396.112 ± 87.3	0.007
Albumin (g/L)	32.56 ± 5.4	30.9 ± 4.11	0.009
HBsAg positive (n)	30	41	–
HBV DNA (log_10_ copies/mL)	4.44 ± 1.25	4.16 ± 1.62	0.024
Creatinine (μmol/L)	70.63 ± 26.9	79.65 ± 37.6	0.011
Prothrombin time (s)	24.1 ± 6.6	26.4 ± 9.8	0.044
International normalized ratio	2.31 ± 0.57	2.77 ± 0.55	0.056
IL-1β(pg/ml)	1.49 ± 0.63	1.57 ± 0.87	0.78
IL-18(pg/m)	287.33 ± 85.9	355.27 ± 100.14	0.007

*Abbreviations*: *ACLF* acute-on-chronic liver failure, *IL-1β*, interleukin-1β, *IL-18*, interleukin-8

Patients with hepatitis A, hepatitis C, hepatitis D, hepatitis E, Epstein-Barr virus, cytomegalovirus, or human immunodeficiency virus were excluded. The study was approved by the medical ethics committee of Beijing Youan Hospital, Capital Medical University, and written informed consent was obtained from each patient. The procedures followed were in accordance with the ethical standards of the responsible committee on human experimentation and with the Helsinki declaration of 1975, as revised in 1983.

### Measurement of serum levels of caspase-1

The levels of caspase-1 in the human serum were measured using a commercially Human Caspase-1/ICE Quantikine ELISA Kit according to the manufacturer’s instructions (R&D systems, Minneapolis, MN, USA). The serum levels of caspase-1 were determined in duplicate in serum aliquots that had undergone 1 or 2 freeze-thaw cycles.

### Measurement of serum cytokine levels using a chemiluminescent immunoassay

Using a commercially available MILLIPLEX MAP Human Cytokine/Chemokine Kit (Millipore, Billerica, MA, USA), we measured the serum levels of inflammasome-associated cytokines, including interleukin 1β (IL-1β) and IL-18, according to the manufacturer’s instructions.

### Measurement of hepatic caspase-1 activity

In order to determine the activity of caspase-1 in the liver tissue of human subjects, liver homogenates were made in lysis buffer and analysed using a colorimetric caspase-1 assay kit (catalogue no: C1102, Beyotime company, China) according to the manufacturer’s instructions.

### Quantitative reverse-transcription polymerase chain reaction

Total RNA was isolated from liver tissue using TRIzol reagent according to the manufacturer’s protocol. Then a total of 1 μg RNA was reverse-transcribed into cDNA using Prime Script First Strand cDNA Synthesis Kit (TaKaRa Bio, Inc., Otsu, Japan). The final reaction of quantitative PCR was in a mixture of 20 μl, composed with 10 μl SYBR Green (TaKaRa Bio, Inc.), 4 μl cDNA, 0.4 μl each primer (10 μM), and 5.2 μl diethylpyrocarbonate water, performed with a quantitative PCR instrument (ABI Prism 7500; Applied Biosystems Inc. Waltham, MA, USA). The reaction condition was 50 °C for 2 min, 95 °C for 5 min, then 95 °C for 15 secs, and 60 °C for 30 secs, for 40 cycles, and then 41 cycles of 55 °C for 4 secs. The mRNA levels were calculated using the 2^–△△Ct^ method with the hypoxanthine phosphoribosyl transferase (HPRT) gene as control [16].

### Immunofluorescence staining

Liver cryosections were fixed by cold methanol followed by permeabilization with 0.1% Triton X100 in PBS. For single staining, after the sections were blocked for 20 min in 10% goat serum in PBS, they were incubated overnight at 4 °C with the caspase-1 mouse monoclonal antibody specific for an epitope mapping between amino acids 367–391 near the C-terminus of caspase-1 of human origin (1:200; Santa Cruz Biotechnology, INC, CA, USA). The slides were then incubated with goat anti-mouse IgG H&L (Alexa Fluor® 647) (Abcam, Cambridge, MA, USA) for 45 min. The images were examined on a Nikon Eclipse E800 fluorescence microscope (Nikon Corp., Tokyo, Japan).

### Statistical analysis

The results for continuous variables are expressed as the mean ± standard deviation. Categorical variables are expressed as numbers and percentages. All the data were analysed using SPSS (Statistical Package for the Social Sciences) software version 13.0 (SPSS Inc., Chicago, IL, USA). Comparisons between groups were performed using Kruskal-Wallis analysis of variance (ANOVA), a distribution-free test. Correlations between variables were evaluated using the Spearman rank correlation test. For all tests, 2-sided *P* < 0.05 was considered significant.

## Results

### Demographic and clinical data

Demographic and clinical characteristics of the different serum study groups are shown in Table 1. There were significant differences in albumin, total bilirubin (TBIL), alanine aminotransferase (ALT), aspartate aminotransferase (AST), prothrombin time, international normalized ratio (INR), creatinine, and HBV DNA levels among the groups. Moreover, the differences between IL-1β and IL-18 were also found among the 5 groups, although no significant differences between age and gender were observed.

For ACLF patients, we collected 30 survivors who fulfilled the criteria for spontaneous recovery and 41 non-survivors who died and fulfilled the criteria for non-spontaneous recovery. The clinical features of ACLF patients with survivors or non-survivors are summarized in Table 2.

### Hepatic mRNA and protein expression of caspase-1 in HBV-related liver disease

**Fig. 1:**
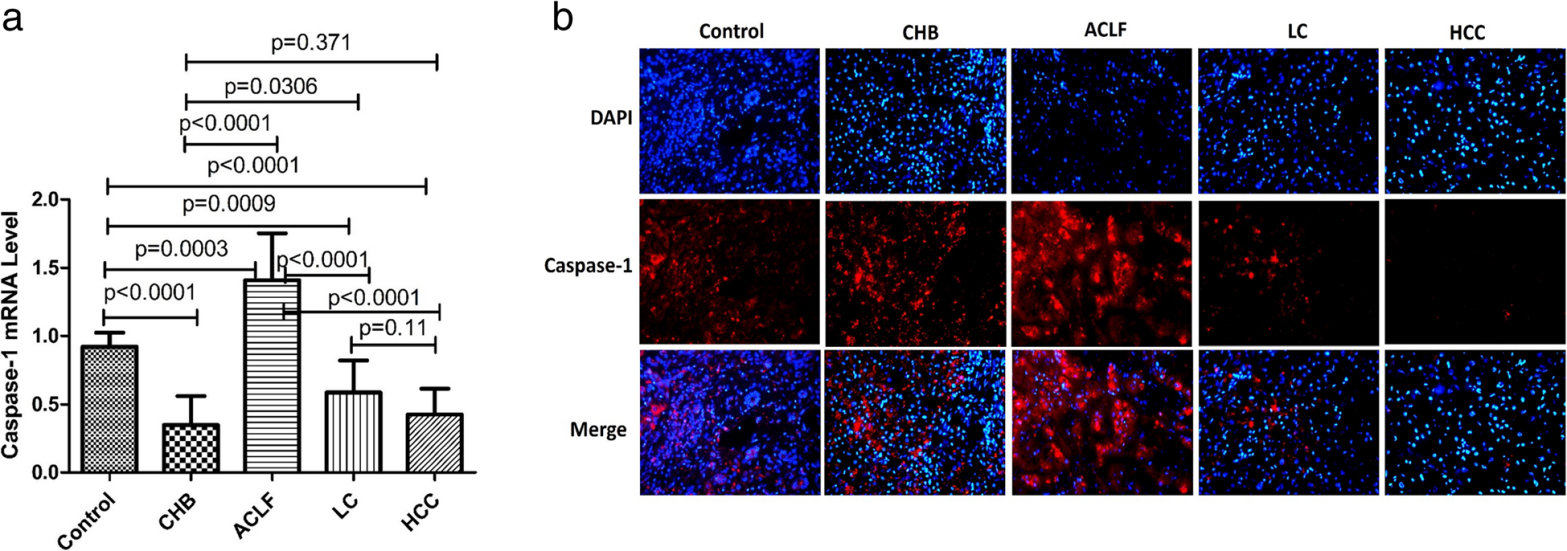
Hepatic mRNA and protein expression of caspase-1 in HBV-related liver disease. **a** Gene expression of caspase-1 was measured by quantitative real-time PCR in normal subjects, CHB patients, LC patients, HCC patients and ACLF patients. **b** The protein levels of caspase-1 were measured with immunofluorescent assays in normal subjects, CHB patients, LC patients, HCC patients and ACLF patients (200×)

First, we measured hepatic the mRNA and protein levels of caspase-1 in HBV-related liver disease. Compared with normal subjects, the qRT-PCR results showed that the relative mRNA levels of caspase-1 were downregulated in CHB patients, LC patients, HCC patients, but upregulated in ACLF patients (Fig. 1a). Furthermore, the results of the immunofluorescent assay showed that the protein levels of caspase-1 were increased in ACLF patients and decreased in HCC patients and LC patients and were slightly increased in CHB patients compared with normal subjects (Fig. 1b). Thus, the hepatic level of caspase-1 is distinctively regulated in CHB, ACLF, LC and HCC patients caused by HBV infection.

### Serum caspase-1 levels and hepatic caspase-1 activity in HBV-related liver disease

**Fig. 2:**
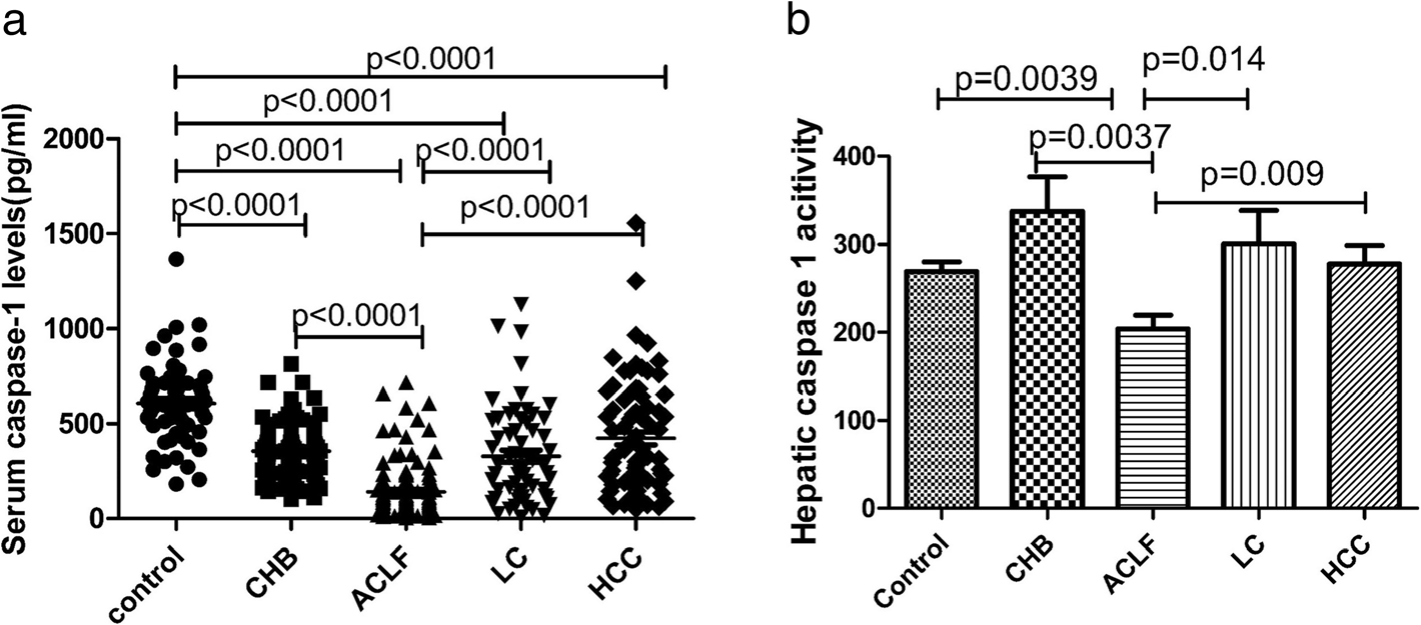
Serum levels of caspase-1 levels and hepatic caspase-1 activity in HBV-related liver disease. **a** Serum levels of caspase-1 in normal subjects and in CHB, ACLF, LC and HCC patients. **b** Activity of caspase-1 in the liver of normal subjects, CHB, ACLF, LC and HCC patients

We further measured the serum levels and hepatic activity of caspase-1 in CHB, ACLF, LC and HCC patients. The serum levels of caspase-1 in CHB, ACLF, LC and HCC were significantly decreased compared with normal subjects, and the serum level of caspase-1 in ACLF was the lowest among the four patient groups (Fig. 2a). Regarding the hepatic activity of caspase-1, there were significant increases only in CHB patients compared with the normal control group, and there were no significant differences among the LC, HCC and normal control groups; furthermore, the hepatic activity was decreased in the ACLF group compared with the control CHB, LC and HCC groups (Fig. 2b). Thus, these findings suggested that the serum level and hepatic activity of caspase-1 are decreased in ACLF.

### Serum levels of caspase-1 in ACLF patients, including survivors and non-survivors

**Fig. 3:**
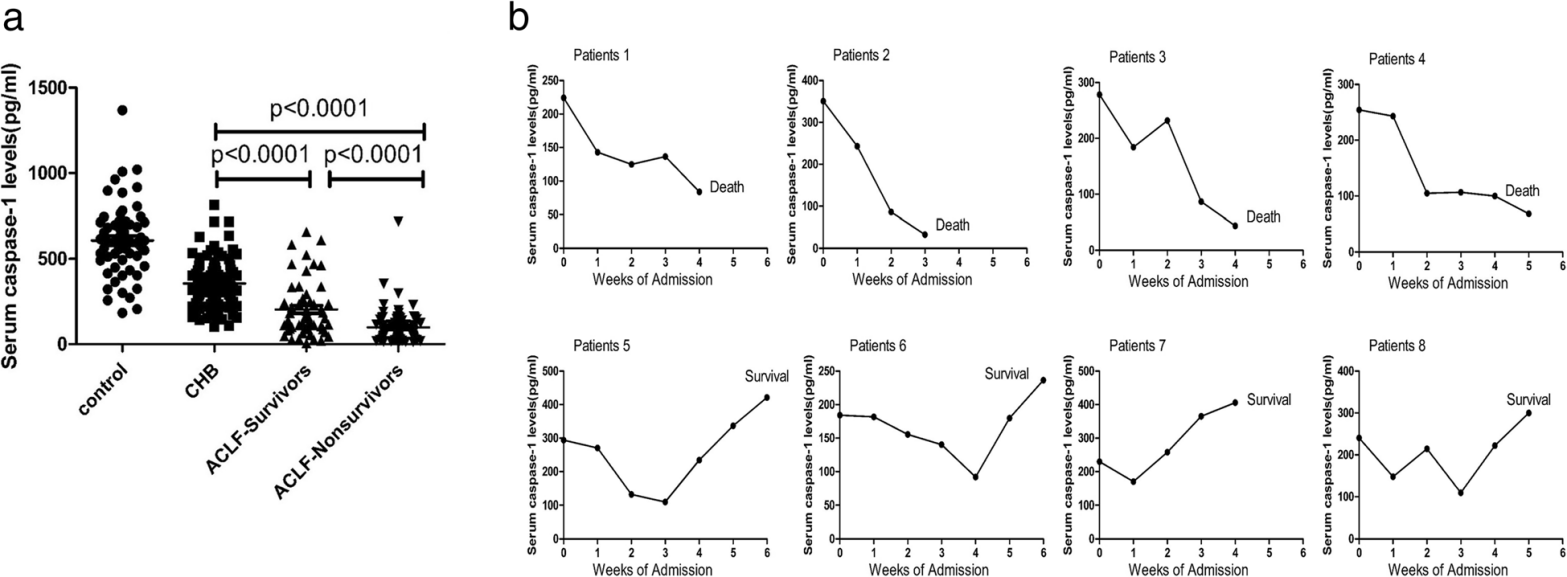
Serum levels of caspase-1 in ACLF patients. **a** Serum caspase-1 levels in normal subjects, ACLF survivors and ACLF non-survivors. **b** Dynamic serum caspase-1 levels of eight ACLF patients, including four who died and four who survived

Based on the above findings, the following study focuses on the caspase-1 levels in ACLF patients caused by acute exacerbation of CHB. We measured the serum levels of caspase-1 in the liver of CHB and ACLF patients including survivors and non-survivors. The serum caspase-1 levels in ACLF-survivor patients were much higher than in ACLF-non-survivor patients (Fig. 3a). We further dissected the association between dynamic serum caspase-1 levels and different prognoses of representative ACLF patients, including four decedents and four survivors. The serum caspase-1 levels exhibited a marked reduction in ACLF patients; however, the caspase-1 levels of all survival patients were rebounded again, while they consistently declined in all death patients during the progression of ACLF (Fig. 3b). These data clearly revealed a correlation between the kinetics of caspase-1 and the development prognosis of ACLF.

### Association of serum caspase-1 levels with liver injury in ACLF

**Fig. 4:**
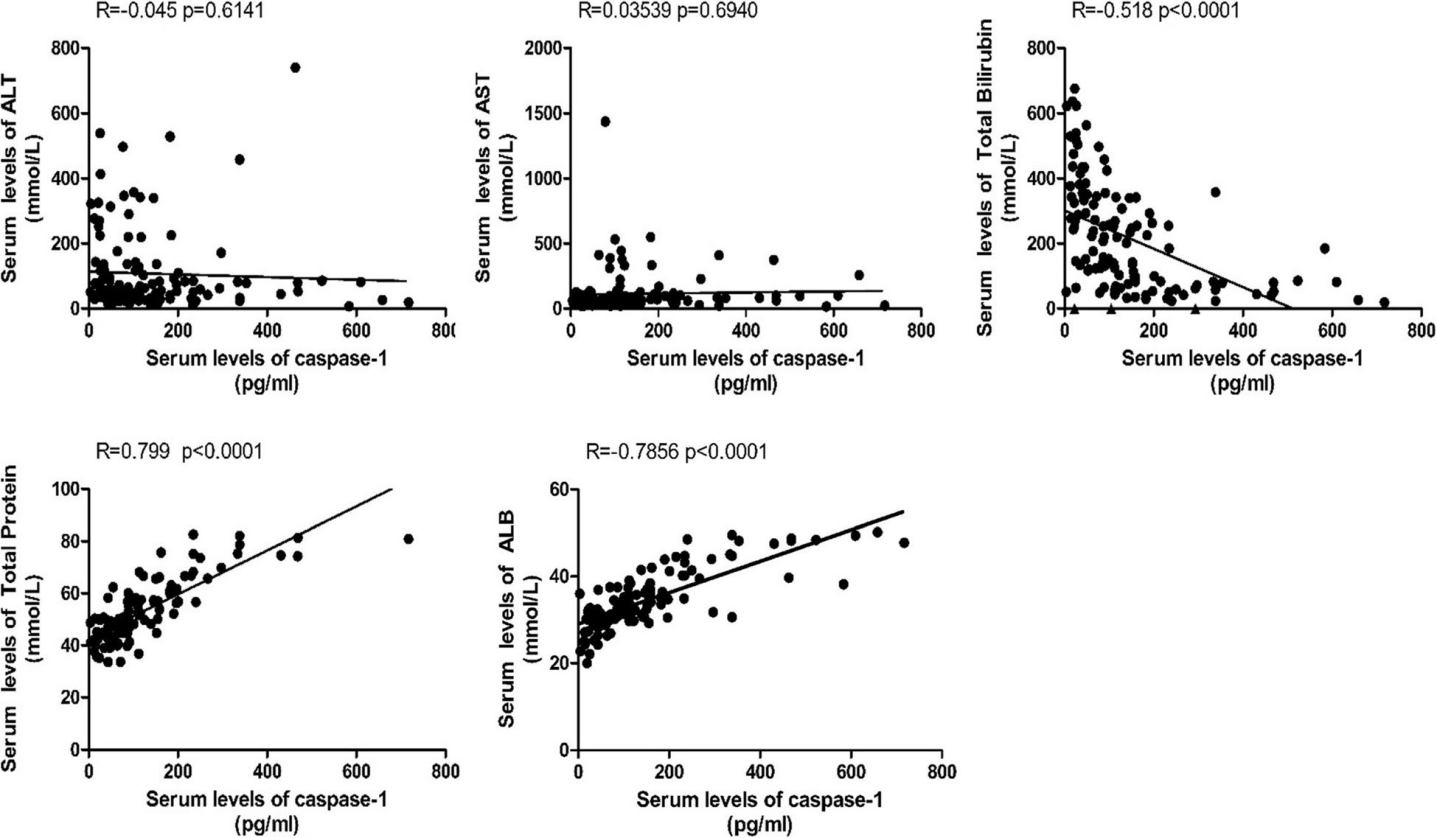
Association of serum caspase-1 levels with liver injury in ACLF. The correlation between serum caspase-1 levels and liver injury in ACLF subjects by Pearson correlation analysis was assessed, including serum ALT levels, AST levels, total bilirubin (Tbil) levels, albumin (ALB) levels and total protein (TP) levels

We next assessed the possible correlation between serum caspase-1 levels and liver injury in ACLF subjects. Pearson correlation analysis showed that there were no significant correlations between serum ALT levels, AST levels and serum caspase-1 levels, but total bilirubin (Tbil) levels had significant negative correlations, and albumin (ALB) levels and total protein (TP) levels had significant positive correlations with serum caspase-1 levels (Fig. 4). Thus, these data show that the serum level of caspase-1 is gradually depressed during the progression of acute exacerbation of CHB, and its level has a negative correlation with liver injury.

### Potential diagnostic and prognostic value of caspase-1 for ACLF

**Fig. 5:**
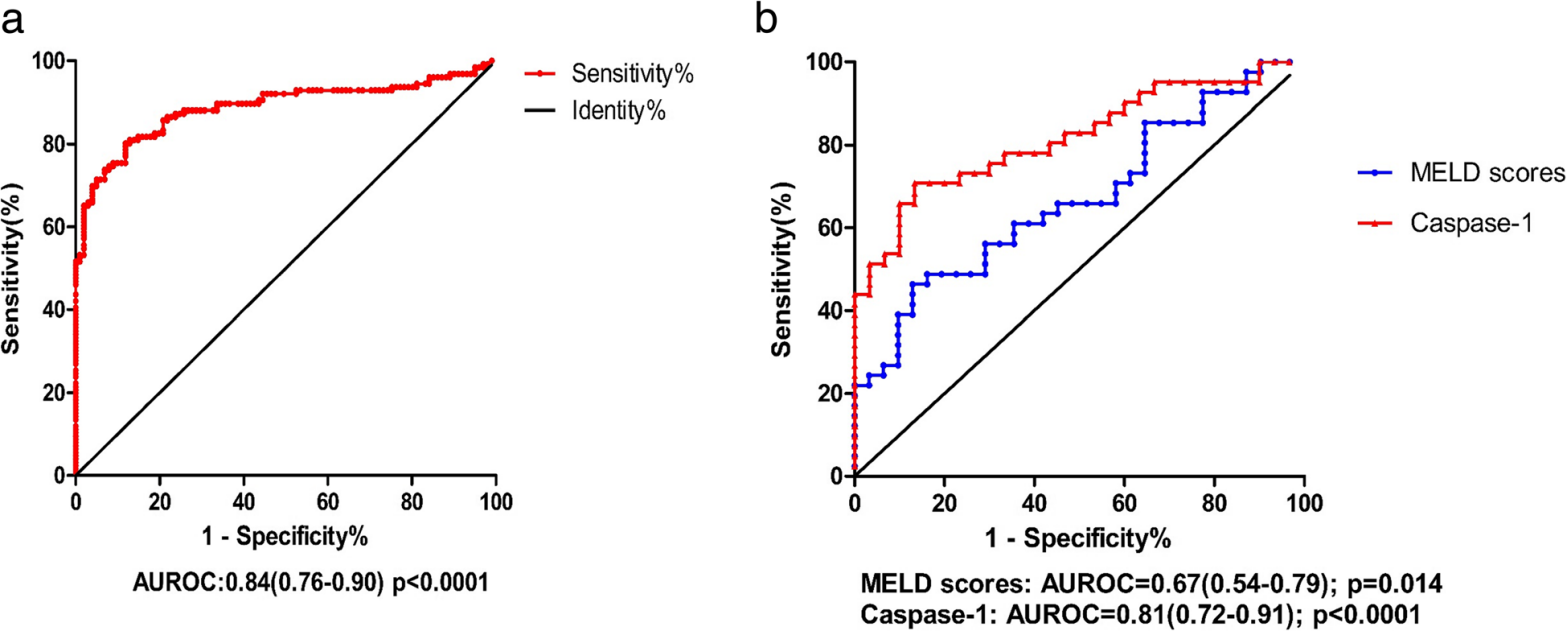
Diagnostic and prognostic value potential of caspase-1 for ACLF. **a** Serum caspase-1 levels show good diagnostic performance in identifying ACLF patients from CHB patients. **b** Comparison of the prognostic values of MELD scores and serum caspase-1 levels in ACLF patients

Receiver operating characteristic (ROC) curves were constructed to evaluate whether caspase-1 could serve as a diagnostic and prognostic biomarker of ACLF. Serum caspase-1 exhibited a high accuracy in discriminating ACLF from CHB patients, with an area under the ROC curve (AUROC) of 0.89 (95% CI 0.76–0.90, *P* < 0.0001) (Fig. 5a). The sensitivity and specificity of caspase-1 for ACLF diagnosis were 80.95 and 87.13%, respectively, in which the cut-off value was set at 203.3 ng/ml. Thus, circulating caspase-1 is a potential diagnostic biomarker for ACLF. To investigate the possible prognostic value of caspase-1 in surviving and non-surviving ACLF patients (detailed in Table 2), we conducted an AUROC analysis to compare the prognostic performance of caspase-1 and MELD scores. The AUROC of caspase-1 (0.81, 95% CI: 0.72–0.91) was higher than those of the MELD (0.67, 95% CI: 0.54–0.79) (Fig. 5b). Thus, the serum levels of caspase-1 may be a potential diagnostic and prognostic value potential of caspase-1 for ACLF.

## Discussion

The inflammasome is an important part of the natural immune system and can be used as a therapeutic target for anti-infective treatment of various inflammatory diseases [17]. HBV infection can progress to asymptomatic carrier status or to CHB, LC, HCC or ACLF, and the inflammasome plays a complex role in different stages of HBV-related liver disease. The inflammasome induces the maturation of pro-inflammatory cytokines, including IL-1β and IL-18, to participate in the innate immune response and liver tissue homeostasis. HBeAg inhibits inflammasome activation and the production of pro-inflammatory cytokines such as IL-18, thus inhibiting the antiviral immune response of the body, facilitating the chronic HBV infection state [18]. Liver fibrosis can be regulated by inflammasomes directly and indirectly [19]. The direct regulation mainly occurs through inflammasome expression in hepatic stellate cells (HSCs) to promote hepatic fibrosis, and the indirect pathway for liver fibrosis is regulated by inducing IL-1β and IL-18 secretion in hepatic macrophages to promote HSC activation [20, 21]. For HCC, the NLRP3 inflammasome components are lost or significantly downregulated in liver cancer tissues, and the greater the deficiency of inflammasomes, the more advanced the cancer progression is [22]. For HCC prognostication, the research demonstrated that the high expression of NLRP3, NLRC4, and caspase-1 in background non-tumorous liver is negatively correlated with patient survival after resection of HCC [23].

In this paper, we first found that the hepatic and serum levels of caspase-1 are differentially expressed among ACLF, LC and HCC patients who developed CHB, which is able to distinguish ACLF from LC and HCC. The hepatic expression of caspase-1 in CHB, ACLF, LC and HCC was compared. We found that caspase-1 shows a distinctive expression profile in different prognoses from CHB: compared with normal subjects, caspase-1 is downregulated in LC and HCC patients, whereas caspase-1 is upregulated in ACLF patients. According to the above interesting results, we can conclude that caspase-1 plays different roles in the pathogenesis of different prognoses derived from CHB. More significantly, there was the opposite trend between the serum levels and hepatic activity levels of caspase-1: compared with the normal control, the serum levels of caspase-1 were reduced in CHB, ACLF, LC and HCC patients and lowest in ACLF patients; in addition, the hepatic activity of caspase-1 was also lower in ACLF patients than in any of the other groups. Based on the above results, we naturally assume that caspase-1 may be differentially regulated in HBV-related end-stage liver disease.

Another novel finding in this paper is that the measurement of caspase-1 serum levels can early diagnosis and predict survival and death of HBV-related ACLF. According to the definition of ACLF by the Asian Pacific Association for the Study of the Liver (APASL), ACLF is distinct from liver cirrhosis and is caused by hepatitis B in the Asia-Pacific region. ACLF mortality is associated with organ failure and systemic inflammation [24, 25], which limits the current evaluation system, such as the Child-Pugh and Model for End-Stage Liver Disease (MELD) scores and their variants. Furthermore, due to the difference of aetiologies and the heterogeneity of existing definitions of ACLF between the Asia-Pacific region and Europe/North America, there is a call to explore other markers of prognosis that could cover the two central prognostic determinants and define outcome with better accuracy regardless of the aetiologies [26]. In our study, all ACLF cases caused by acute insult were based on CHB, which were devoid of decompensated cirrhosis. It is very doubtful that the hepatic levels of caspase-1 mRNA and protein were high while the serum level of caspase-1 was low in ACLF patients. The possible reason for this difference was that the expression of caspase-1 was increased during the development of ACLF. If the morphology of hepatocytes is intact, the synthesized caspase-1 cannot be released into the blood, leading to low levels of caspase-1 in serum. If the many hepatocytes are necrotic in the late stage of ACLF, the synthesized caspase-1 will be reduction, and the level of caspase-1 in serum will be decreased. In addition, the liver tissue in our study originated from patients in the early stage of ACLF, at which time there were no too many necrotic hepatocytes; however, for the serum of ACLF, there were come from patients in different stages of injury (including early, middle and late stages). Therefore, the above two reasons may explain the difference that a higher level in the liver and a lower level in the serum, but all these possibilities need further study.

More importantly, the expression levels of serum caspase-1 in HBV-related ACLF patients also indicate the survival and death of patients. As the first caspase identified in mammalian cells, caspase-1 not only plays an important role in the inflammatory response but also triggers pyroptosis, a newly discovered programmed cell death of inflammatory cells. Furthermore, the caspase-1 serum level has a significant relation to the indicators that can reflect liver function and the overall status of patients, such as ALT, AST, and total protein. Thus, we believe that caspase-1 may be an effective biomarker for the diagnosis and prognosis of HBV-related ACLF. However, the early diagnosis and prognostic evaluation value of caspase-1 in HBV-related ACLF patients requires further validation of large sample studies.

## Conclusions

In conclusion, our study demonstrated that caspase-1, one of the critical components of the inflammasome, plays a critical role in the pathogenesis of ACLF derived from CHB; moreover, the serum level of caspase-1 may act as a non-invasive biomarker for early diagnosis and prognosis of ACLF caused by acute exacerbation of CHB.

## Data Availability

The datasets used and/or analyzed during the current study are available from the corresponding author on reasonable request.

## Abbreviations

ACLF: Acute-on-chronic liver failure
ALB: Albumin
ALT: Alanine aminotransferase
AST: Aspartate aminotransferase
AUROC: Area under the ROC curve
CHB: Chronic hepatitis B
D-GalN: D-Galactosamine
HBV: Hepatitis B virus
HCC: Hepatocellular cancer
HPRT: Hypoxanthine phosphoribosyl transferase
IL-1β: Interleukin-1β
INR: International normalized ratio
LC: Liver cirrhosis
LPS: Lipopolysaccharide
MELD: Model for End-Stage Liver Disease
PAGE: Polyacrylamide gel electrophoresis
ROC: Receiver-operating characteristic
TBIL: Total bilirubin
TP: Total protein
